# Revolutionizing Concrete: Performance Enhancement and Elemental Insights with Electric Arc Furnace (EAF) Slag Replacement

**DOI:** 10.3390/ma18071528

**Published:** 2025-03-28

**Authors:** Jing Cheng Jason Ting, Foo Wei Lee, Kim Ho Yeap, Ren Jie Chin, Ming Kun Yew, Chun Chieh Yip

**Affiliations:** Department of Civil Engineering, University Tunku Abdul Rahman, Sungai Long Campus, Jalan Sungai Long, Bandar Sungai Long, Kajang 43000, Selangor, Malaysia; leefw@utar.edu.my (F.W.L.); yeapkh@utar.edu.my (K.H.Y.); yewmk@utar.edu.my (M.K.Y.); yipcc@utar.edu.my (C.C.Y.)

**Keywords:** electric arc furnace slag, compressive strength, interfacial transition zone, carbon sequestration, concrete durability

## Abstract

This study explores the influence of Electric Arc Furnace (EAF) slag particle size and replacement percentage on the engineering performance of concrete, providing valuable insights into its optimal utilization for sustainable construction. By analyzing particle size ranges—R1 (0.8–2.36 mm), R2 (2.36–4.75 mm), and R3 (4.75–7.0 mm)—this research highlights their distinct contributions to compressive strength and carbonation potential. Medium-sized particles (R2) emerged as the most suitable due to consistent compressive strength across different replacement percentages, high calcium content, and superior carbonation efficiency, leading to the highest calcium carbonate formation and CO_2_ uptake. The novelty of this work lies in integrating advanced analytical techniques, including Scanning Electron Microscopy (SEM) and Energy Dispersive X-ray Spectroscopy (EDX), to elucidate the microstructural mechanisms driving these performance enhancements. The findings establish a quantifiable relationship between EAF slag’s high calcium and magnesium oxide content and its role in mechanical improvements and carbon dioxide sequestration via mineral carbonation reactions, with R2 achieving the highest CO_2_ uptake. This comprehensive approach addresses the apparent contradiction between early-stage and long-term performance, emphasizing R2’s suitability, with 45% of the replacement of fine aggregate as the optimal choice for sustainable high-performance concrete with superior strength stability and carbonation efficiency.

## 1. Introduction

Concrete is globally recognized as the most widely used construction material, prized for its durability, versatility, and cost-effectiveness. However, conventional concrete production is associated with significant environmental impacts, including resource depletion and greenhouse gas emissions. As the construction industry faces increasing pressure to adopt sustainable practices, it is imperative to explore alternative materials that can mitigate these concerns while maintaining or even enhancing the mechanical and durability properties of concrete [1].

Fine aggregates, predominantly composed of sand, play a crucial role in traditional concrete mixes, contributing to volume, workability, and mechanical strength. However, the extraction of natural sand has led to severe environmental issues, such as resource depletion and ecosystem disruption. To mitigate these challenges, industrial byproducts like Electric Arc Furnace (EAF) slag have emerged as promising alternatives, offering both mechanical performance enhancements and environmental benefits, such as carbon capture [1].

EAF slag, a byproduct of steel production, contains high levels of oxides such as calcium (CaO), magnesium (MgO), and silicon (SiO_2_), which contribute to its pozzolanic and carbonation potential. Studies have shown its ability to improve concrete compressive strength and durability while reducing reliance on Portland cement, thus lowering CO_2_ emissions [2]. However, research gaps remain in understanding the specific mechanisms by which EAF slag influences concrete performance, particularly the interplay of particle size, replacement level, and carbonation efficiency.

For instance, research has demonstrated that EAF slag can enhance compressive strength and reduce the need for Portland cement, thus lowering CO_2_ emissions [3]. However, gaps remain in understanding the specific mechanisms by which EAF slag affects concrete performance, especially regarding the optimal levels of replacement and particle size. This study aims to investigate the effect of different EAF slag replacement levels (15%, 30%, and 45%) and particle sizes (R1, R2, and R3) on the compressive strength of concrete, and to elucidate the underlying mechanisms. By clarifying these relationships, this research contributes valuable insights into the use of EAF slag in concrete production [4].

The experimental methodology includes compressive strength tests, Scanning Electron Microscopy (SEM), and Energy Dispersive X-ray Spectroscopy (EDX) to validate the effectiveness of EAF slag replacement. The compressive strength test will assess the mechanical performance of concrete at varying slag replacement levels, while SEM will analyze microstructural changes, revealing how particle sizes affect the bonding within the concrete matrix. EDX will provide elemental composition analysis, helping us to understand the chemical interactions that influence concrete properties [5].

The chemical composition of EAF slag, which includes high levels of calcium oxide (CaO) and silicon dioxide (SiO_2_), makes it beneficial for the hydration process, contributing to both mechanical strength and carbon sequestration potential. The slag was sieved into three particle size ranges—R1 (0.8–2.36 mm), R2 (2.36–4.75 mm), and R3 (4.75–6.30 mm)—to study the effects of particle size on concrete performance [6].

Research has shown that EAF slag can significantly contribute to carbon dioxide (CO_2_) neutrality in concrete. A study published in the *Journal of CO_2_ Utilization* [7] reviews the potential of steelmaking slags, including EAF slag, for CO_2_ sequestration through carbonation, highlighting its effectiveness in binding CO_2_ into stable mineral phases. Additionally, studies such as the role of EAF slag is emphasized in reducing the carbon footprint of concrete while improving durability [2].

Recent studies highlight Electric Arc Furnace (EAF) slag’s potential in sustainable concrete applications, emphasizing its role in enhancing mechanical properties and reducing the environmental impact of traditional cement. Research on different types of EAF slag, particularly oxidizing and reducing slags, reveals that their chemical composition—including elements like Fe, Ca, Si, Mg, and Al—and physical characteristics such as particle size and density significantly affect concrete performance [8]. For instance, the variability in slag properties based on source and processing conditions influences compressive strength, density, and durability. Studies show that EAF slag can improve concrete’s compressive strength and increase resistance to chemical attacks, especially when used in specific particle sizes and replacement ratios. Furthermore, EAF slag contributes to lower carbon emissions, offering additional environmental benefits through its CO_2_ sequestration potential during hydration and carbonation processes [9].

Moreover, a detailed analysis of EAF slag’s chemical composition reveals significant influences on concrete performance, particularly through oxides like CaO, SiO_2_, Al_2_O_3_, MgO, and Fe_2_O_3_. High calcium oxide (CaO) content, commonly found in oxidizing EAF slags, is advantageous for concrete, as it contributes to the formation of calcium silicate hydrate (C-S-H), which is essential for early-age strength development. This increased CaO content enhances the binding matrix, correlating with higher initial compressive strengths [10].

Silicon dioxide (SiO_2_), meanwhile, plays a critical role in improving long-term strength and durability. As SiO_2_ gradually reacts with calcium hydroxide during hydration, it fills pore spaces and densifies the matrix, especially in reducing slags that have higher SiO_2_ levels. This contributes to enhanced resistance to chemical attack and long-term stability. Aluminum oxide (Al_2_O_3_) content also significantly affects strength development, where higher levels often result in faster setting times and early strength gains, albeit with a potential reduction in workability. Additionally, Al_2_O_3_ can influence ettringite formation, which has implications for long-term durability. Magnesium oxide (MgO), though present in smaller quantities, plays a role in volume stability. Excess MgO can cause delayed expansion due to hydration after setting, potentially impacting durability if not carefully controlled [11]. However, optimal MgO levels support matrix densification without risking expansion, contributing to stable strength development. Iron oxide (Fe_2_O_3_), more prevalent in reducing EAF slags, improves durability by enhancing resistance to chloride penetration, which indirectly benefits structural longevity. While Fe_2_O_3_ does not directly increase compressive strength, it helps protect embedded reinforcements, ultimately supporting concrete’s durability and extending its lifespan. This oxide-specific understanding underscores how variations in EAF slag composition contribute to differing concrete performance outcomes, emphasizing the importance of optimizing chemical compositions to achieve targeted strength and durability [12].

For instance, the study in *ISIJ International* [13] explores the mineralogical and chemical transformation of steel slag, emphasizing its implications for long-term stability and reactivity. Similarly, the research published in *Cement and Concrete Composites* [14] examines the interaction of slag-based systems with CO_2_, providing critical insights into carbonation mechanisms and microstructural evolution. Integrating these references will help position the current study within the broader scope of research on steel slag utilization and provide a robust foundation for the proposed methodology and analysis.

This study addresses these gaps by comprehensively evaluating the effects of different EAF slag particle size ranges (R1: 0.8–2.36 mm, R2: 2.36–4.75 mm, and R3: 4.75–6.30 mm) and replacement levels (15%, 30%, and 45%) on the mechanical and environmental performance of concrete. Advanced analytical techniques, including Scanning Electron Microscopy (SEM) and Energy Dispersive X-ray Spectroscopy (EDX), were employed to investigate the microstructural and elemental mechanisms, particularly within the interfacial transition zone (ITZ), that drive these enhancements.

The findings demonstrate a clear relationship between EAF slag particle size and concrete performance, with smaller particles (R1) enhancing long-term durability through increased pozzolanic reactivity, and larger particles (R3) excelling in early strength development due to rapid hydration and ITZ densification. Medium-sized particles (R2) provide a balance, making them suitable for versatile applications. Furthermore, this study highlights the role of EAF slag in carbon sequestration through mineral carbonation reactions, linking its chemical composition—especially high calcium and magnesium oxide content—to its ability to enhance mechanical properties while capturing CO_2_.

The novelty of this research lies in its holistic approach to optimizing EAF slag utilization by integrating microstructural analysis and carbonation efficiency. Unlike prior studies, this work quantifies the interplay between particle size, replacement level, and microstructural transformations, providing actionable insights for tailoring EAF slag configurations to specific structural applications. This study ultimately contributes to advancing low-carbon high-performance concrete technologies, aligning immediate strength gains with long-term sustainability objectives.

Moreover, this study leverages insights from the recent literature, such as the mineralogical transformations in steel slag in *ISIJ International* [13] and its carbonation interactions (*Cement and Concrete Composites*) [14] to position its findings within the broader context of sustainable concrete development. By linking the results to practical applications and balancing immediate strength gains with long-term sustainability benefits, this paper highlights the transformative potential of EAF slag in advancing low-carbon concrete technologies [10].

## 2. Materials

### 2.1. Raw Materials

#### 2.1.1. EAF Slag

The Electric Arc Furnace (EAF) slag utilized in this study was procured from regional steel production facilities in Malaysia. This slag, a byproduct of steel manufacturing, underwent a comprehensive characterization process to evaluate its suitability and performance-enhancing potential for concrete applications. To optimize the performance of the slag as a replacement material, the EAF slag was sieved using a standardized sieve analysis method. The sieving process classified the particles into three size ranges to investigate their impact on concrete properties [15].

For a description of the EAF slag used in this research, chemical composition of the EAF slag is calcium oxide (CaO): 40%, a critical component contributing to cementitious reactions and carbonation potential; silicon dioxide (SiO_2_): 30%, enhances the durability and strength of the concrete matrix through pozzolanic reactions; magnesium oxide (MgO): 8%, contributes to volume stability and densification of the concrete matrix; iron oxide (Fe_2_O_3_): 10%, improves resistance to chloride penetration and structural durability; and trace elements (12%), including compounds such as aluminum oxide (Al_2_O_3_), manganese oxide (MnO), and sulfur trioxide (SO_3_), which may influence hydration and strength development.

For particle size distribution, R1: Concrete specimens consist of steel slag within a range of between 4.75 mm and 7.0 mm. R2: Concrete specimens consist of steel slag within a range of between 2.36 mm and 4.75 mm. R3: Concrete specimens consist of steel slag within a range of between 0.80 mm and 2.36 mm.

Building on the established knowledge of Supplementary Cementitious Materials, SCMs, this study explores the novel use of Electric Arc Furnace (EAF) slag as a replacement for fine aggregates, expanding the scope of sustainable practices in concrete production. While traditional SCMs primarily modify the binder phase, EAF slag contributes to the aggregate phase, offering unique advantages in terms of particle size distribution, chemical composition, and carbonation potential. By studying the interaction of EAF slag with the cement matrix, this research seeks to complement existing SCM knowledge and provide insights into its dual role in enhancing mechanical performance and contributing to carbon sequestration [5].

The variability in the chemical composition of Electric Arc Furnace (EAF) slag based on particle size significantly influences the reproducibility of both the mechanical and chemical properties of concrete in real-world applications. Mechanical properties such as compressive strength and flexural strength are particularly sensitive to particle size distribution [16]. Smaller particle sizes (0.80–2.36 mm) offer a higher surface area, enhancing bonding with the cement matrix and promoting early strength development, but excessive replacement or poor distribution may result in increased porosity, compromising strength. Conversely, larger particles (4.75–7.0 mm) contribute to aggregate interlock, which enhances tensile and compressive performance, although their lower specific surface area may limit chemical reactivity.

The chemical properties of concrete incorporating EAF slag also vary with particle size. Smaller particles exhibit higher carbonation efficiency due to increased surface area, which facilitates greater CO_2_ uptake during mineralization. Larger particles, while less reactive, may exhibit slower carbonation rates, potentially affecting long-term performance. Moreover, particle size impacts the leachability of trace elements, raising concerns about environmental safety and the material’s long-term durability. Finer particles tend to accelerate hydration and pozzolanic reactions, contributing to early strength gains, whereas coarser particles provide bulk density but less chemical reactivity [17].

These variations present challenges to the reproducibility of concrete properties, particularly when scaling from laboratory conditions to real-world applications. Material heterogeneity and the difficulty of maintaining consistent particle size distribution in large-scale production can result in performance variability. Additionally, environmental factors such as temperature and humidity further amplify these inconsistencies. To address these issues, the standardized characterization of EAF slag’s chemical composition across particle sizes is essential. Optimizing the blending of different particle size ranges can balance mechanical strength, workability, and durability. Furthermore, extensive field trials are necessary to evaluate performance under real-world conditions, and pre-treatment methods such as milling or heat treatment could homogenize the slag’s properties. These strategies are critical to ensuring the reliable and reproducible use of EAF slag in concrete for sustainable construction.

#### 2.1.2. Cement

The primary binder used in this study was Ordinary Portland Cement (OPC), meeting ASTM C150 specifications for Type I cement. OPC was selected due to its well-established mechanical and chemical compatibility with supplementary materials like Electric Arc Furnace (EAF) slag. Detailed properties and chemical composition of the cement will be described as below.

For a description of the cement used in this research, the chemical composition of cement consists of calcium oxide (CaO): 62.0%, a critical component for the formation of calcium silicate hydrates (C-S-H), which impart strength; silicon dioxide (SiO_2_): 30.0%, responsible for secondary pozzolanic reactions that enhance long-term strength and durability; aluminum oxide (Al_2_O_3_): 5.0%, influencing early strength development and workability; and iron oxide (Fe_2_O_3_): 3.0%, improving the cement’s resistance to sulfate attack.

Certification of cement: The OPC was sourced from a reputable supplier and certified for compliance with ASTM C150 and Malaysian Standards MS EN 197-1. The physical properties are Blaine Fineness: 300 m^2^/kg, ensuring sufficient surface area for effective hydration, and Specific Gravity: 3.15, contributing to the calculation of mix proportions.

#### 2.1.3. Aggregates

The fine aggregates used in this study were river sand obtained from local suppliers. The sand was selected for its consistency and compliance with industry standards, ensuring its suitability for concrete production.

The source of aggregate used in this research, which is natural river sand, was sourced locally to ensure availability and sustainability. The grading of the sand conformed to the specifications outlined in ASTM C33, which defines requirements for the grading and quality of fine aggregates used in concrete. Compliance with ASTM C33 guarantees the aggregates’ appropriateness for structural applications.

For physical properties, Specific Gravity: 1560 kg/m^3^. This parameter indicates the sand’s density and plays a vital role in calculating mix proportions for achieving the desired workability and strength in concrete.

#### 2.1.4. Water

The water used in this study was locally sourced in Malaysia and was selected to meet the requirements for concrete mixing and hydration processes.

For the description of water used in this research, the water is locally sourced from Malaysia and conformed to the ASTM C1602 standard for mixing water in concrete, ensuring that it was suitable for maintaining the quality and consistency of the mix.

### 2.2. Mixture Proportions and Specimen Preparation

**Figure 1:**
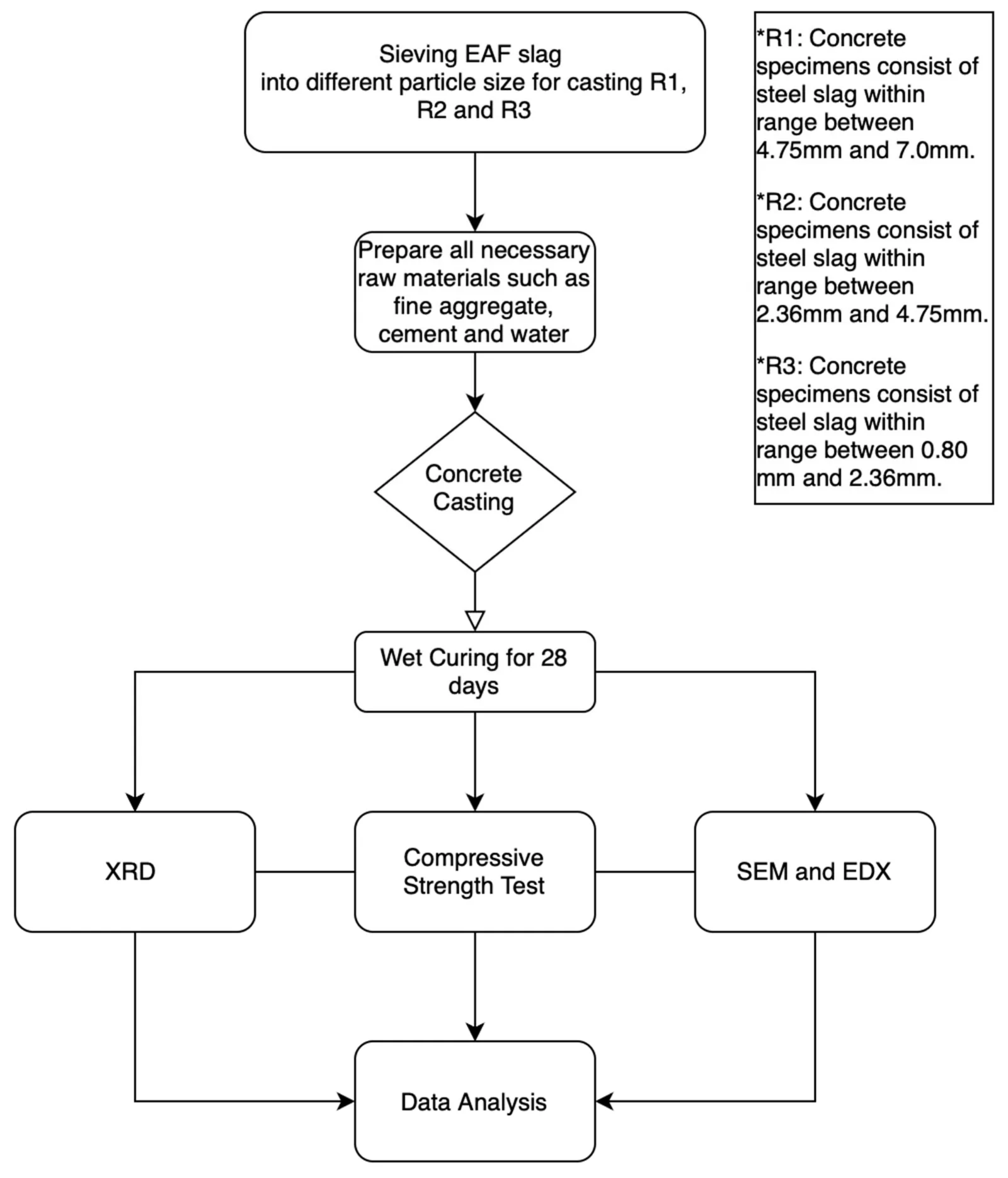
Flow Chart.

**Figure 2:**
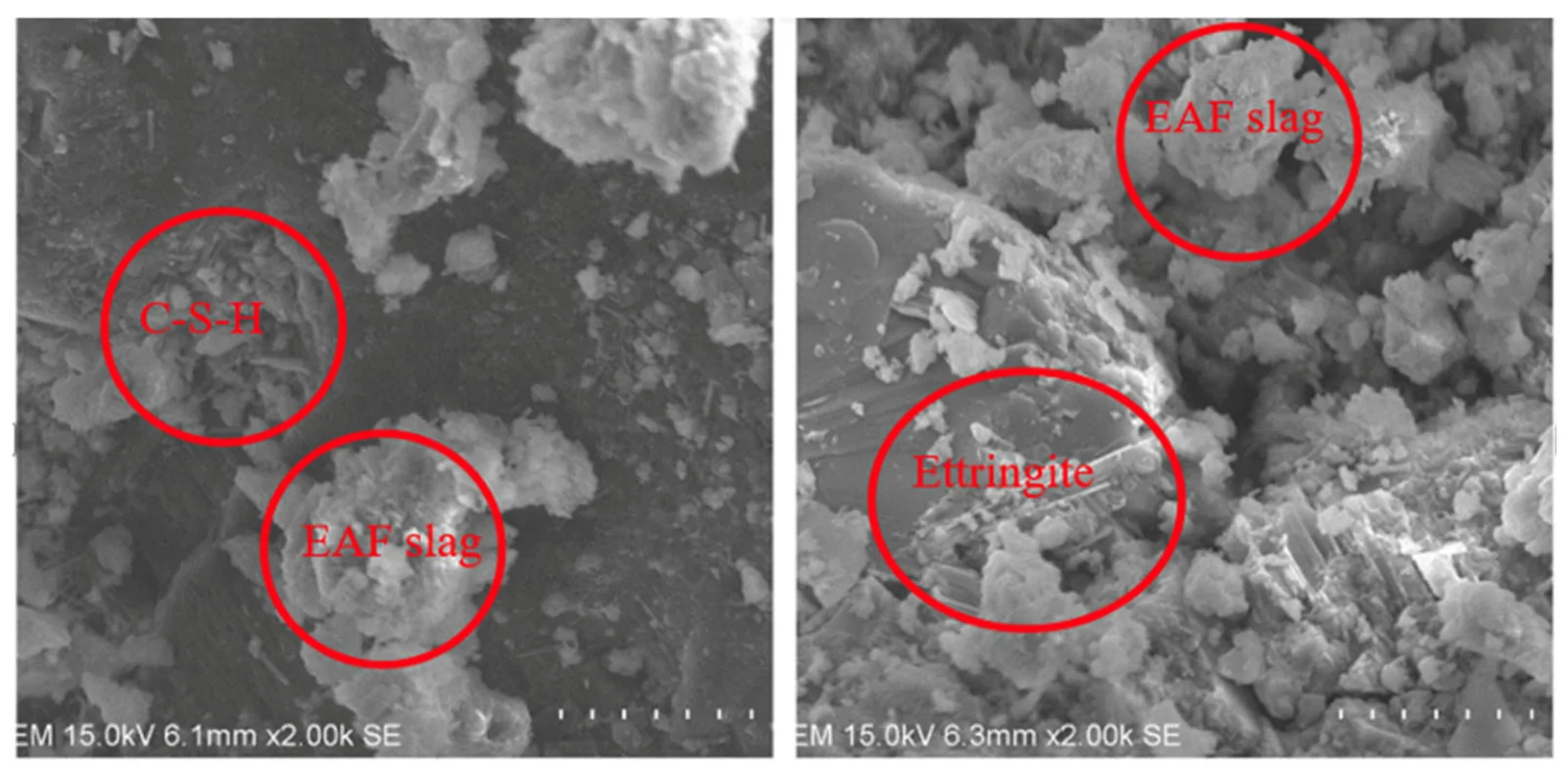
SEM images of formation of calcium silicate hydrate (CSH), CaCO_3_.

In this study, a detailed procedure is presented in the flowchart shown in Figure 1. a water–cement ratio of 0.55 is used, and cement and sand are used in equal amounts. The quantity of raw materials is increased by 30% to allow for mixing-stage waste. The absolute volume method is used to calculate the percentage of each material. Four different proportion mixes were prepared, with the control set presented in Table 1. One of the proportion mixes, incorporating 15% EAF replacement, is shown in Table 2. The SEM image of the materials is depicted in Figure 2. EAF slag primarily consists of magnesium, calcium, and silica, which can form as calcium carbonate with formula of CaO + CO_2_ = CaCO_3_.

The concrete mixing process was carried out using a mechanical mixer following a systematic procedure. Initially, the dry materials, including cement, EAF slag, and fine aggregates, were added to the mixer. These components were thoroughly mixed for five minutes to achieve a homogeneous blend. Subsequently, water was gradually introduced into the dry mix, and the mixing process continued for an additional 2–3 min to ensure the uniform distribution and proper hydration of the concrete mixture.

## 3. Results

Laboratory testing such as compression tests, X-ray diffraction (XRD), Scanning Electron Microscopy (SEM), and Energy Dispersive X-ray Spectroscopy (EDX) were conducted to collect necessary data on the chemical properties, physical properties, and mineral composition of the samples.

### 3.1. Compressive Strength Test

The compressive strength test was conducted using standard 150 mm concrete cubes to evaluate the mechanical properties of concrete mixtures with varying levels of Electric Arc Furnace (EAF) slag replacement. A total of 30 samples were prepared and tested, ensuring sufficient data to calculate average results and minimize variability in assessing the influence of different particle sizes and replacement levels on the overall strength of the concrete.

The test was conducted using a universal compression testing machine that adheres to ASTM C39 standards. Concrete cubes with standard dimensions of 150 mm × 150 mm × 150 mm were prepared, cured, and tested after 28 days.

The load was applied gradually at a constant rate of 0.2 MPa per second until failure occurred. The maximum load at failure was recorded, and the compressive strength was calculated by dividing this load by the cube’s cross-sectional area (22,500 mm^2^). The average compressive strength from at least three specimens was reported, including details on mix design, specimen age, curing conditions, and any anomalies observed.

### 3.2. X-Ray Diffraction (XRD)

XRD was employed to examine the crystalline structure of the samples, revealing the atomic arrangement and mineral composition. The crystalline structure of the samples was analyzed using a PANalytical X’Pert PRO MPD X-ray diffractometer (Almelo, The Netherlands). The machine was configured with a Cu Kα radiation source (wavelength: 1.54060 Å), operating at 40 kV and 30 mA. The scan range was set between 5° and 85° (2θ), with a step size of 0.02° and a scan speed of 2°/min. This setup provided thevprecise identification of mineral phases in the samples.

### 3.3. Scanning Electron Microscopy (SEM)

High-resolution microstructural images of the samples were obtained using a JEOL JSM-7600F Field Emission Scanning Electron Microscope (FE-SEM) (Tokyo, Japan). The SEM operated at an acceleration voltage of 10–15 kV, with a working distance of 8 mm. Sample preparation included polishing and coating with a thin layer of gold using a sputter coater to enhance conductivity and image clarity.

### 3.4. Energy Dispersive X-Ray Spectroscopy (EDX)

Elemental composition analysis was conducted using an Oxford Instruments X-Max 80 EDX Detector integrated with the JEOL JSM-7600F SEM system (Tokyo, Japan). EDX detector was calibrated using standard reference materials, providing a resolution of 125 eV at Mn Kα.

The primary elements of interest included iron (Fe), calcium (Ca), silicon (Si), magnesium (Mg), and aluminum (Al), which are crucial for assessing the slag’s suitability for carbon capture and other applications. The analysis parameters included [specific parameters], ensuring the precise measurement of elemental concentrations.

## 4. Discussion

The data collected from the experiments conducted in the laboratory are summarized in tables and graphs to facilitate an easier understanding of the results. This chapter is concerned with presenting the results and an explanation of the experiments conducted, such as EDX, XRD, SEM, and a compression test to evaluate the carbon capture potential, the optimum particle size range, and compressive strength of the EAF slag concrete samples. These tests help in providing necessary information on the EAF slag to determine its potential in achieving the aim and objectives of this study.

SEM tests produce highly magnified images that can reach a magnification of up to 300,000 times and even up to 1,000,000 times to provide the sample’s detailed information such as size, shape, composition, and crystallography. The image produced helps the researchers obtain a clearer picture of the internal structure of the samples. The EDS test is always used along with the SEM test for determining the elemental composition of solid-state materials at the micrometer scale [7].

XRD analyzes the crystal structure of materials. By shining X-rays through a sample, XRD measures the angles and intensities of the X-rays scattered by the sample’s atomic structure. This information provides insights into the atomic or molecular arrangement within materials, aiding in the identification of crystal structures and the study of defects or imperfections [10].

Compressive strength is one of the important parameters of the mechanical properties of concrete as well as tensile strength, flexural strength, shear strength, and modulus of elasticity. The compressive strength of concrete is the only mechanical property that is studied due to the limitation and scope of this study.

### 4.1. Compressive Strength Test Results

**Figure 3:**
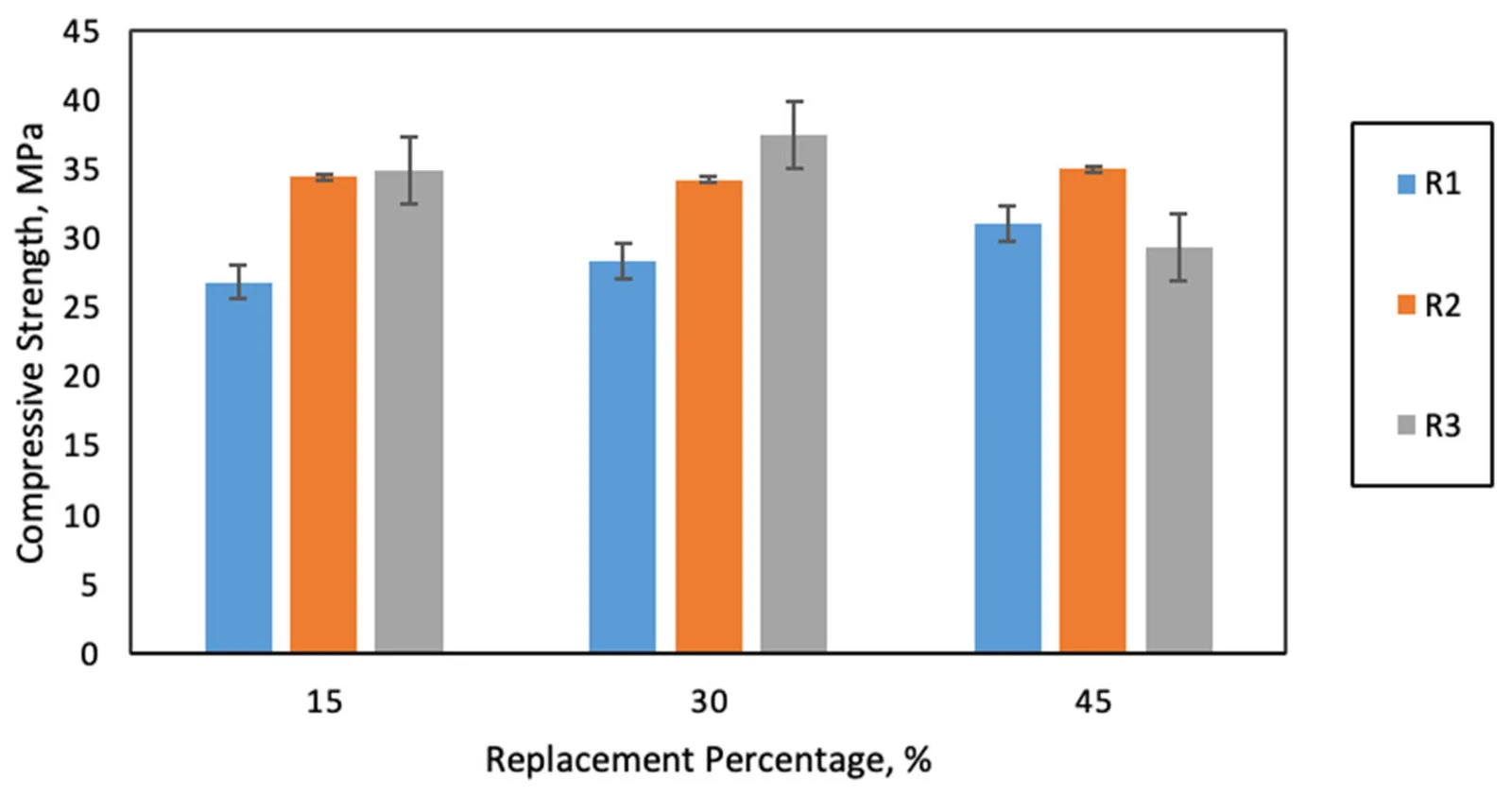
Average compressive strength of EAF slag concrete under different particle size ranges and percentages of replacement.

The results of the compressive strength tests shown in Table 3 and Figure 3 highlight the influence of EAF slag particle size and replacement percentage on the mechanical performance of concrete. The compressive strength of concrete generally increased with larger particle sizes and higher replacement percentages. Among all categories, R3 (4.75–7.0 mm) at 30% replacement achieved the highest compressive strength of 37.48 MPa, slightly surpassing R2 (2.36–4.75 mm), which reached 34.25 MPa. However, at 45% replacement, R3 experienced a strength reduction, whereas R2 maintained its performance, achieving 35.04 MPa, making it the most suitable option for consistent mechanical strength.

This trend suggests that larger EAF slag particles contribute to better mechanical interlocking and enhanced load transfer within the cement matrix. The angularity and rough surface texture of the slag improve the interfacial transition zone (ITZ), leading to stronger bonding with the cement paste. While R3 provided the highest strength at 30% replacement, its strength dropped at 45%, indicating potential limitations in maintaining long-term mechanical stability. In contrast, R2 demonstrated consistent strength gains across different replacement levels, highlighting its reliability for structural applications.

Smaller particles (R1: 0.8–2.36 mm) exhibited slightly lower initial compressive strength, but showed potential for long-term durability. The increased surface area of R1 particles promoted hydration and pozzolanic reactions, enhancing microstructural density over time. While R1 may not provide the highest immediate strength, its contribution to long-term performance makes it suitable for applications requiring extended service life.

Higher replacement percentages consistently resulted in increased compressive strength, reflecting the beneficial incorporation of EAF slag into the concrete matrix. The slag’s high calcium content played a crucial role in reducing void spaces, creating a denser and more cohesive matrix. These findings align with previous studies, such as a report resulting that EAF slag aggregates improve compressive strength due to their rough texture and high Specific Gravity [9]. Similarly, research by [14] confirmed that concrete with EAF slag replacement exhibited superior stiffness and mechanical integrity compared to traditional mixes.

The superior early performance of R3 particles makes them ideal for applications requiring immediate strength, such as structural load-bearing components. Their higher calcium and magnesium content facilitates rapid hydration and carbonation, leading to early strength gains. Additionally, R3’s increased surface area enhances ITZ bonding, reducing porosity and strengthening the microstructure. However, the decline in strength at 45% replacement suggests that excessive R3 content may impact long-term stability.

In contrast, R2 emerged as the most suitable sample due to its consistent compressive strength across different replacement levels. With the highest calcium content (82.2%) and balanced silica content (12.77%), R2 supports both early and long-term strength development. Its carbonation potential also enhances durability, making it particularly advantageous for environmentally driven applications. The ITZ in R2 concrete facilitates CO_2_ diffusion, improving carbonation efficiency and contributing to environmental sustainability, making it the optimal choice for applications requiring both structural reliability and long-term durability.

### 4.2. SEM and EDX

**Figure 4:**
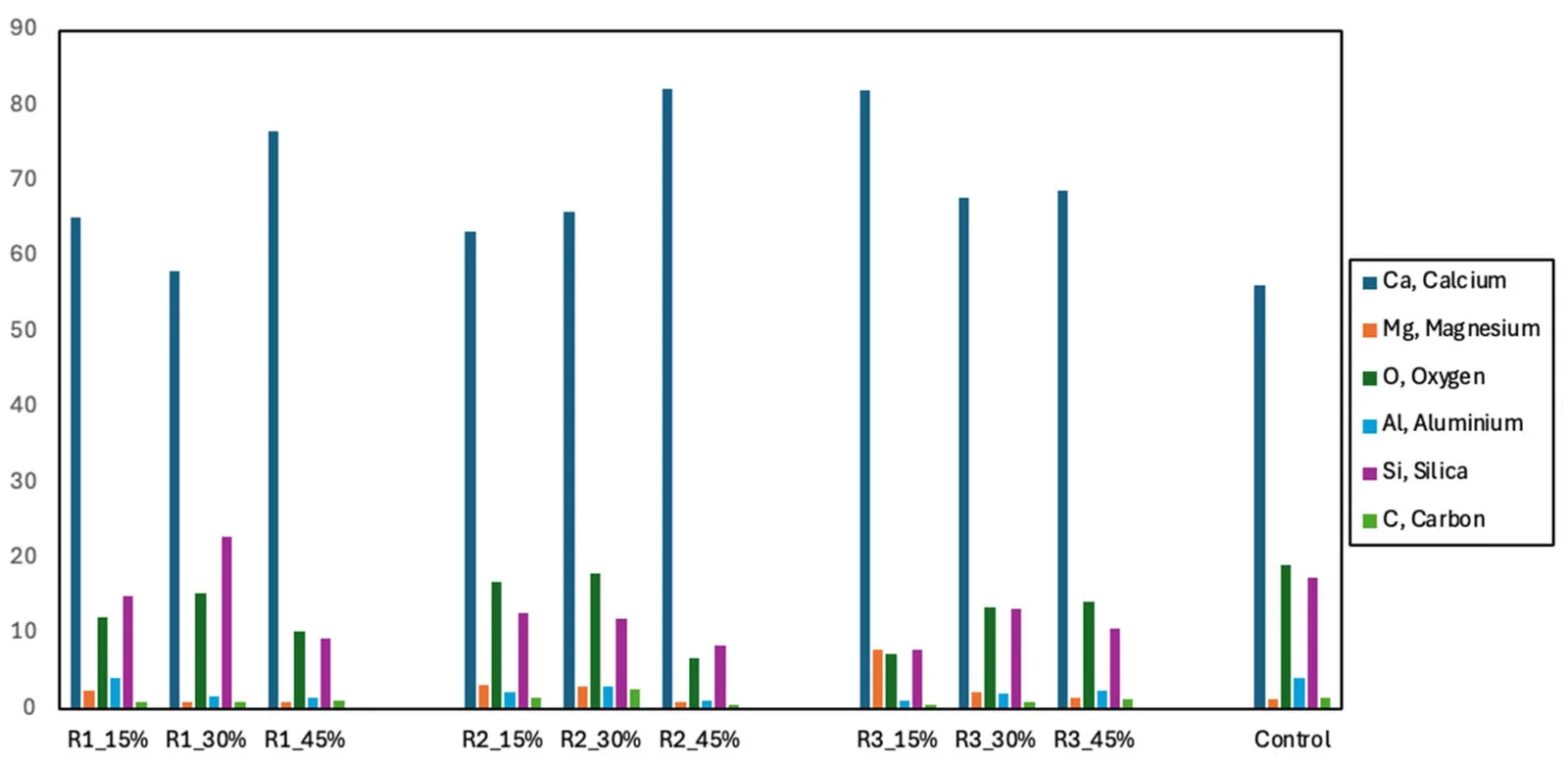
Element percentage of EAF across different particle size ranges (R1, R2, and R3).

Through the observation data shown in Figure 4 and Table 4 Medium-sized particles (R2: 2.36–4.75 mm) emerged as the most suitable sample due to their balanced chemical composition, consistent compressive strength, and superior calcium content. R2 contained the highest calcium content (82.2%) and a significant silica content (12.77%), which contributed to both early and long-term strength development. The compressive strength for R2 concrete at 45% replacement was the highest among all particle sizes, reaching 35.04 MPa. This strong mechanical performance can be attributed to enhanced hydration and carbonation reactions, ensuring structural integrity over time. Furthermore, R2 exhibited the highest carbon content (1.54%), indicating its superior carbonation potential, which enhances its role in CO_2_ sequestration.

Smaller particles (R1: 0.8–2.36 mm) demonstrated a higher silica content (14.92%) and greater surface area, which enhanced their reactivity during hydration. This promoted the formation of calcium silicate hydrate (C-S-H), a critical compound for early strength development. Additionally, the higher silica levels facilitated pozzolanic reactions, improving long-term strength and durability. These reactions played a key role in densifying the concrete matrix and increasing resistance to environmental degradation. The results indicated that R1 concrete with 45% replacement achieved a compressive strength of 31.09 MPa, confirming its ability to maintain durability while improving carbon sequestration potential.

Larger particles (R3: 4.75–7.0 mm) exhibited high calcium (82.04%) and magnesium (7.84%) content, making them suitable for immediate strength development. The compressive strength of R3 concrete at 30% replacement reached 37.48 MPa, the highest among all particle sizes tested. However, at 45% replacement, R3 experienced a decline in strength, dropping to 32.67 MPa. The lower silica content (7.84%) in R3 meant that its pozzolanic reactivity was less pronounced, reducing the secondary formation of calcium silicate hydrate (C-S-H). Additionally, its carbonation potential was lower than R2, which further affected long-term durability. This suggests that, while R3 enhances early strength due to its mechanical interlocking, its lower silica content and weaker ITZ at higher replacement levels contribute to strength loss over time.

Overall, R2′s high calcium content, superior carbonation efficiency, and consistent compressive strength make it the most suitable choice for concrete applications requiring both durability and environmental sustainability. While R3 exhibited the highest compressive strength at 30% replacement, its drop in performance at 45% replacement, as revealed by SEM and EDX analyses, highlights potential long-term stability issues. R2, on the other hand, maintained strength stability, reaching 35.04 MPa at 45% replacement, making it the optimal choice for applications prioritizing both structural reliability and CO_2_ sequestration.

### 4.3. X-Ray Diffraction (XRD) Results

X-ray diffraction is another analytical technique that is commonly used for studying the crystallinity of materials using X-rays. X-ray examination is performed by packing the sample with a stream of X-rays. A diffraction pattern of the sample is then seen, and the interpretation value provides information on how the atoms are organized or packed within the material. X-ray diffraction is used for the identification of the crystals of compounds, the measurement of their purity, and research on phase changes or structural conversion in a variety of environmental conditions. Furthermore, it is used to analyse atomic bonding and detect impurities oe deficiencies with high precision, making it essential for studying nanomaterials of significant importance such as steel slag mineralization. In this study, the setting of X-ray diffraction (XRD) follows the setup of using a gonio scan axis, which is configured with specific parameters to ensure accurate data collection. The scan range is set and fixed at a range of 5.0000° to 85.0000° 2θ, with a fine step size of 0.0200° 2θ. The scan step time is set as 1.0000 s and a 0.0000° 2θ offset is maintained for alignment accuracy. Meanwhile, the utilization of a pre-set time scan type ensures the consistency of data acquisition across samples. Beam divergence during measurements is controlled using the fixed divergence slit type with a size of 1.0000°. The sample length is limited to 10.00 mm with a receiving slit size of 0.1000 mm to optimize the detection of diffracted X-rays. Operation at a stable measurement temperature of 25.00 °C ensures reproducibility in experiments. The XRD system utilizes a Cu anode material with characteristic wavelengths of K-Alpha1 at 1.54060 Å and K-Alpha2 at 1.54443 Å, which provides essential information for accurate data analysis.

**Figure 5:**
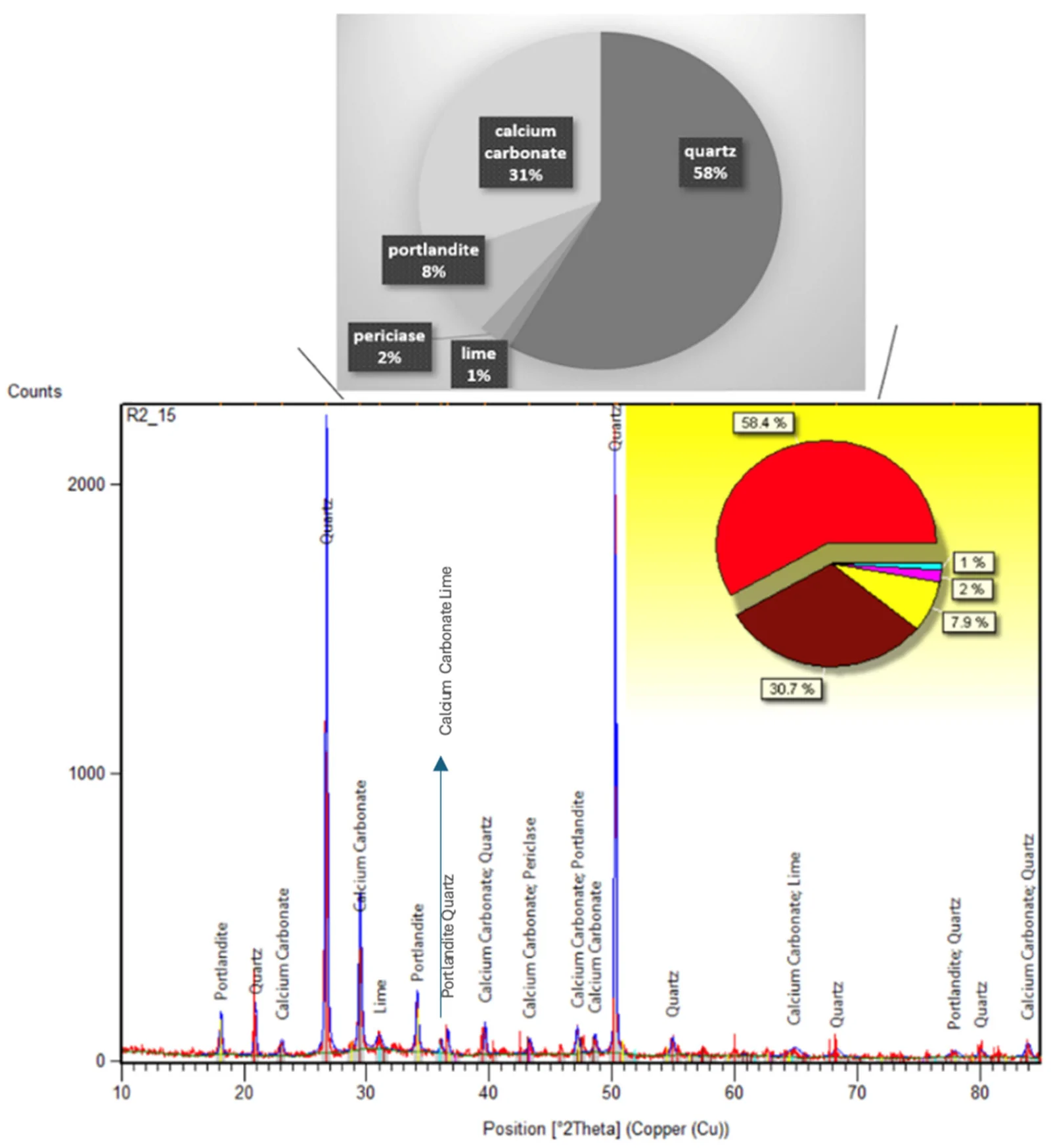
XRD graph of R2 15% of concrete sample: 15% representing a replacement percentage of steel slag with sand aggregate during concrete production, and R2 representing a medium particle size range (2.36 mm to 4.75 mm).

By referring to the XRD graph in Figure 5, the X-ray diffraction (XRD) analysis of the concrete specimen (R2) composition by using X-pert HighScore Plus software in version 5.1 as the analysis tool, which was prepared with a 15% replacement of fine sand by steel slag with particle sizes ranging from 2.36 mm to 4.75 mm, revealed several key phases in the material. The dominant phase was identified as quartz, which accounted for 58.4% of the total composition. This mineral, commonly found in sand and aggregates, contributes significantly to the strength and durability of the concrete.

Calcium carbonate, representing 30.7% of the composition, was also detected. This phase likely resulted from the carbonation process, or it may have originated from limestone used in the concrete mix. While calcium carbonate can contribute to the concrete’s strength, its presence might also indicate carbonation, a process that can lead to the corrosion of steel reinforcement over time.

Portlandite, which is a by-product of the cement hydration process, was identified and accounted for 7.9% of the composition. This phase plays a crucial role in maintaining the high pH of the pore solution, thereby protecting the steel reinforcement from corrosion. Additionally, periclase, a phase typically associated with steel slag, was observed in small quantities (2%). The incorporation of steel slag into the concrete was confirmed by the presence of this phase. However, it should be noted that excessive periclase could potentially lead to undesirable expansion due to the formation of magnesium hydroxide (brucite) during hydration.

Lime was also detected, albeit in a small proportion (1%). This phase, produced during the hydration process, can contribute to the early strength development of the concrete, but may pose durability issues if not properly managed over time.

The presence of portlandite (Ca(OH)_2_) and periclase (MgO) as key phases in the Electric Arc Furnace (EAF) slag concrete can have significant implications for its long-term expansion and durability. Portlandite, while contributing to the alkalinity of the pore solution and aiding in carbonation reactions, can be a source of vulnerability under sulphate-rich environments. When exposed to sulphates, portlandite reacts to form gypsum and ettringite, which can lead to expansion and cracking, compromising structural integrity. Similarly, periclase, when not fully hydrated, may undergo delayed hydration over time, forming brucite (Mg(OH)_2_). This reaction is accompanied by volumetric expansion, which can cause microcracking, further impacting the durability and longevity of the material.

This study acknowledges the absence of long-term tests, such as sulphate attack resistance and freeze–thaw cycles, which are critical for evaluating the performance of EAF slag concrete under aggressive environmental conditions. Without these tests, it is difficult to fully quantify the comparative durability of EAF slag concrete against conventional concrete. However, the existing literature suggests that the high density and low permeability of well-processed EAF slag concrete may offer better resistance to freeze–thaw cycles by minimizing water ingress and subsequent freeze–thaw damage.

The inclusion of steel slag as a partial replacement for fine sand introduced additional elements, such as periclase, which are not typically found in conventional concrete. While the incorporation of steel slag can enhance the mechanical properties and durability of the concrete, the presence of periclase must be carefully monitored. If not adequately managed, the hydration of periclase to brucite could lead to volume expansion, potentially causing microcracking or long-term durability issues.

It is suggested that the concrete specimen with R2 samples exhibited a stable mineral composition, with quartz and calcium carbonate as the primary phases. The ongoing cement hydration was evidenced by the presence of portlandite. Although the steel slag’s contribution to the mix was confirmed by the presence of periclase, its low quantity indicated controlled reactivity, which should contribute to a durable concrete mix if the expansion potential of periclase is properly managed in the long term. This process facilitates the systematic and accurate collection of data. After carrying out XRD analysis on normal concrete and EAF slag concretes, the chemical compounds of the concretes are summarized in Table 5 below.

According [16] findings, EAF steel slag typically consists of CaO, SiO_2_, and ferrous oxide (FeO) roughly in the percentage of 40%, 17%, and 20%, respectively. Other oxides such as aluminum oxide (Al_2_O_3_), MgO, and manganese (II) oxide (MnO) are also present in concentrations ranging from 0.4% to 10%. Ref. [17] suggest that EAF slags typically contain 10–40% FeO, 22–60% CaO, 6–34% SiO_2_, 3–14% Al_2_O_3_, and 3–13% MgO, respectively. However, the chemical composition of EAF slag concrete shows a significant presence of CaO, MgO, and SiO_2_, but with varying levels of SO_3_, gypsum, quartz, portlandite, and carbon between different concrete samples. The XRD results differ slightly from previous research, with only CaO, MgO, and SiO_2_ being detected, while FeO, Al_2_O_3_, and MnO are conspicuously absent. There are also differences in the percentage composition. However, this variation can be attributed to the analysis of concrete samples containing EAF slag rather than pure EAF slag. The absence of specific compounds in the concrete samples could be attributed to inherent differences between EAF slag concrete samples and their pure EAF slag counterparts. The scarcity of samples is suggested to be another factor that results in varying levels of chemical compounds in each concrete. In the XRD test, only one sample from each concrete category is sent for analysis. This results in a lack of comparative data among each category and leads to less consistent results.

**Figure 6:**
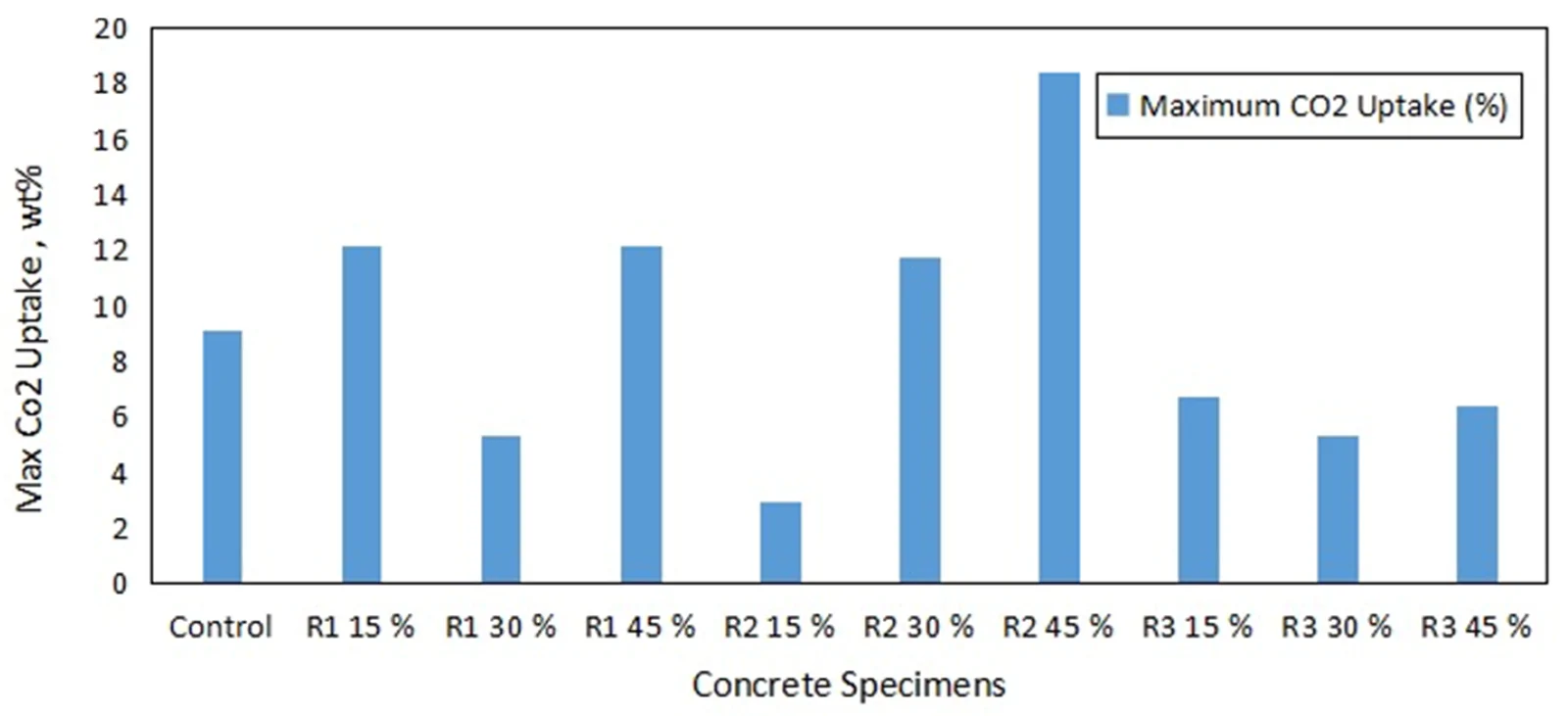
Maximum CO_2_ uptake of various concrete specimens.

The chemical compounds of control set concrete and EAF slag concrete that identified by XRD analysis included calcium oxide (CaO), sulfur trioxide (SO_3_), magnesium oxide (MgO), silicon dioxide (SiO_2_), portlandite (Ca (OH)_2_), gypsum (CaSO_4_·2H_2_O), calcium carbonate (CaCO_3_), and carbon (C). In fact, CaO is a key component in EAF slag concrete that contributes to its carbon capture capacity. During the carbonation process, calcium oxide (CaO) reacts with carbon dioxide (CO_2_) from the atmosphere to form calcium carbonate (CaCO_3_), effectively sequestering CO_2_ within the concrete matrix. A higher CaO content generally indicates a greater potential for carbon capture. Although SO_3_ is not directly involved in carbon capture reactions, its presence can influence the properties of the concrete. Excessive sulfur content can lead to the formation of calcium sulphate (CaSO_4_), which may affect the reactivity of CaO with CO_2_ and potentially reduce the carbon capture capacity. Although MgO can also participate in carbonation reactions, its contribution is lesser compared to CaO. Its presence enhances carbonation kinetics and carbon capture capacity by contributing to the overall alkalinity of the concrete. The chemical compounds discussed above play an important role in affecting the carbon capture capacity of concrete, as the maximum theoretical CO_2_ uptake can be calculated by using the weightage of these chemical compounds such as lime, periclase, sulfur trioxide, calcium carbonate, dipotassium oxide, and disodium oxide. After obtaining the weightage data, the maximum theoretical CO_2_ uptake is calculated using Equation (1), Stenoir’s stoichiometric equation [15]. Since dipotassium oxide and disodium oxide are not found in XRD analysis, Equation (1) will be used to calculate the maximum theoretical CO_2_ uptake. The results are summarized in Table 6 and presented as a clustered bar chart in Figure 6 below.

(1) $${\;\mathrm{CO}}_{2}=0.785\left(\%\mathrm{CaO}-0.7{\;\%\mathrm{SO}}_{3}\right)+1.091\;\%\mathrm{MgO}$$

The calculation of maximum theoretical CO_2_ uptake using Steinar’s stoichiometric equation does indeed simplify the complex chemical processes occurring during carbonation. This approach primarily considers the major compounds (e.g., calcium and magnesium oxides) that react with CO_2_ to form carbonates. However, it excludes the contributions from minor compounds or secondary reactions, such as those involving trace elements or other oxides present in EAF slag. While this simplification is practical for an initial estimation, it may introduce inaccuracies, potentially leading to the over- or under-estimation of the CO_2_ sequestration capacity.

For example, under-estimation could occur if minor compounds such as iron oxides or alumina also participate in carbonation reactions but are not accounted for in the equation. Conversely, over-estimation might result if assumptions about complete conversion of reactive oxides are made, as real-world conditions often prevent full reaction due to kinetic and diffusion limitations, or due to the formation of passivating layers on slag particles.

To mitigate these potential inaccuracies, complementary analytical techniques, such as Energy Dispersive X-ray Spectroscopy (EDX) or X-ray Fluorescence (XRF), can be used to experimentally validate the actual CO_2_ uptake and compare it with theoretical predictions. These methods provide insights into the extent of carbonation and the role of minor compounds, offering a more comprehensive understanding of the carbonation process.

Moreover, sensitivity analyses can be performed to evaluate the impact of excluding minor compounds or secondary reactions on the calculated CO_2_ sequestration capacity. Incorporating correction factors derived from experimental data into the stoichiometric equation can further refine the predictions and align them more closely with real-world outcomes. This integrated approach ensures that the simplifications inherent in Steinar’s equation do not compromise the reliability of the sequestration capacity estimates, providing a robust framework for assessing the potential of EAF slag in carbon capture and storage applications.

Figure 6 and Table 6 shows that the maximum CO_2_ uptake value for normal concrete (control) is 9.10%. Among the EAF concrete mixes, R2 with 45% EAF slag content has the highest maximum CO_2_ uptake value at 18.41%. This is followed by R1 45% and R1 15% EAF slag content at 12.17% and R2 with 30% EAF slag content at 11.76%. Among all the mixes tested, R2 with 45% EAF slag content concrete exhibits the highest carbon capture capacity. The data overall suggest that incorporating higher percentages of EAF slag into concrete mixes enhances their carbon capture capacity. The obtained results show better performance compared with previous studies, which have shown that steel slag can capture up to 5.836 wt. % (58.36 g CO_2_/kg of steel slag) of its theoretical carbon sequestration capacity based on its mass [15]. This is probably because of the presence of calcium oxide (CaO) in EAF slag, which can react with atmospheric CO_2_ during the curing process to form calcium carbonate (CaCO_3_), effectively sequestering carbon dioxide within the concrete matrix. As a result, EAF concrete shows promise as a sustainable building material with the potential to reduce carbon emissions through carbon capture and utilization.

On the other hand, the high levels of calcium carbonate ranging from 26% to 67% found in EAF concrete, as shown in Table 5, may be caused by multiple reasons. The reason behind this may be associated with a direct reaction between calcium ions and carbon dioxide (CO_2_) during the hardening of the concrete elements. Furthermore, it is possible that materials that are rich in calcium carbonate are being added to the concrete mixture during the concrete casting process. The level of CaCO_3_ concentration in concrete significantly increases the amount of CO_2_ being captured. Calcium carbonate is created when ambient CO_2_ enters concrete pores and combines with the calcium hydroxide (Ca (OH)_2_) that is produced during the cement hydration process. In the form of calcium carbonate, this process efficiently sequesters CO_2_ within the concrete matrix. Hence, a cement with a higher concentration of CaCO3 traps more CO_2_, as it facilitates more sites to absorb the gas. Additionally, having high levels of silicon dioxide (3–67%) will also help to raise the alkalinity of the concrete. The highly alkaline environment resulting from silicon dioxide improves the formation of calcium carbonate and sequesters carbon dioxide through carbonation. Furthermore, the hydration reaction between Portland cement also releases another chemical known as portlandite or calcium hydroxide (Ca(OH)_2_) when calcium oxide (CaO) from cement reacts with water. Portlandite does not actively participate in the strength of concrete under compression, but it can facilitate this process. Through the process of carbonation, carbon dioxide is effectively captured and completely sequestered, keeping it out of the atmosphere. This can help mitigate global warming by decarbonizing concrete production while also making concrete more sustainable.

Therefore, calcium carbonate (CaCO_3_), silicon dioxide (SiO_2_), and portlandite also have the potential for carbon capture within the concrete matrix. CaCO_3_ can be formed directly by the carbonation of CaO, and portlandite can react with carbon dioxide from the atmosphere that penetrates concrete to form calcium carbonate, while carbon or CO_2_ can be sequestered by mineral carbonation reactions involving CaO and other reactive compounds in the concrete mix. The presence of these chemical compounds is beneficial for the long-term sustainability of concrete in terms of carbon capture potential.

## 5. Conclusions

This study provides new insights into the role of Electric Arc Furnace (EAF) slag particle size and replacement percentage in enhancing the mechanical and environmental performance of concrete. The findings demonstrate that particle size significantly influences compressive strength and carbonation potential, with each size range offering unique benefits. Smaller particles (R1: 0.8–2.36 mm) enhance long-term durability through increased pozzolanic activity, while larger particles (R3: 4.75–7.0 mm) deliver superior immediate strength. Medium-sized particles (R2: 2.36–4.75 mm) strike a balance, making them suitable for versatile applications. These results establish a clear relationship between particle size and concrete performance, an area previously understudied.

The relationship between the compressive strength results and the discussion on R2′s long-term performance can be clarified by distinguishing between early-stage and long-term strength development, particularly in relation to the interfacial transition zone (ITZ). While R3 exhibits higher early compressive strength due to its finer particle size and enhanced hydration, its long-term stability is affected when used at higher replacement levels. In contrast, R2 demonstrates more consistent compressive strength across various replacement levels, which suggests that its particle size and composition contribute to a well-balanced ITZ. This balanced ITZ structure allows for steady strength development over time, ensuring both immediate performance and long-term durability. Thus, the contradiction is resolved by understanding that R2 maintains a stable strength profile due to its optimized ITZ characteristics, whereas R3, despite its initial strength advantage, may experience a decline in performance at higher replacement levels.

Among the tested samples, R2 (2.36–4.75 mm) emerged as the most suitable particle size for concrete applications, exhibiting consistent compressive strength across different replacement levels. This stability suggests that R2 provides an optimal balance between early-stage strength development and long-term durability, making it ideal for sustainable concrete formulations.

Although R3 (4.75–7.0 mm) demonstrated superior early compressive strength, particularly at a 30% replacement level, its long-term performance showed signs of decline at higher replacement levels (45%). The excessive incorporation of R3 appears to negatively impact the interfacial transition zone (ITZ) over time, potentially reducing durability. However, its ability to enhance early strength makes it well suited for applications requiring immediate load-bearing capacity, such as structural components.

R1 (0.8–2.36 mm), while offering benefits in long-term durability through increased pozzolanic activity and carbonation potential, did not exhibit the same level of compressive strength consistency as R2. This suggests that, while R1 contributes to long-term sustainability, its strength development may not be as predictable across various replacement percentages.

In a nutshell, these findings provide actionable insights for optimizing EAF slag usage in concrete production. By selecting appropriate particle sizes and replacement levels, concrete mixtures can be tailored to meet specific performance needs, balancing early strength gains with long-term durability. This study advances the understanding of EAF slag’s role in sustainable construction and reinforces its potential as an eco-friendly alternative to traditional aggregates in high-performance concrete applications.

## Figures and Tables

**Table 1:** Weight of raw materials (control set).

Type of Raw Materials	Proportion Ratio	Weight (kg)	Density (kg/m^3^)
OPC	1	3.97	1440
Sand	1	3.97	1560
Water	0.55	2.18	1000
Total	2.55	≈ 10.125	

**Table 2:** Weight of raw materials (with 15% EAF slag and 2.36–0.8 mm).

Type of Raw Materials	Proportion Ratio	Weight (kg)	Density (kg/m^3^)
OPC	1	3.878	1440
Sand	0.850	3.297	1560
Water	0.550	2.133	1000
Steel Slag	0.249	0.817	1380
Total	2.649	≈ 10.125	

**Table 3:** Average compressive strength of EAF slag concrete under different particle sizes and percentages of replacement.

	Average Compressive Strength (MPa)			
	Percentage of Steel Slag Replacement (%)			
Particle Size Range	0	15	30	45
R1	-	26.88	28.40	31.09
R2	-	34.45	34.25	35.04
R3	-	34.91	37.48	29.39
Control	24.80	-	-	-

**Table 4:** Element analysis of EAF slag with several replacement percentages.

Element	Replacement Percentage of Steel Slag (%)	Ca, Calcium	Mg, Magnesium	O, Oxygen	Al, Aluminum	Si, Silica	C, Carbon
R1 (0.8–2.36 mm)	15	65.13	2.75	12.14	4.12	14.92	0.94
	30	58.06	0.96	15.42	1.76	22.88	0.92
	45	76.64	0.97	10.31	1.56	9.44	1.08
R2 (2.36–4.75 mm)	15	63.42	3.16	16.91	2.2	12.77	1.54
	30	66.04	3.07	18.08	3.07	12.04	2.61
	45	82.2	0.94	6.74	1.11	8.46	0.54
R3 (4.75–7 mm)	15	82.04	7.84	7.29	1.13	7.84	0.65
	30	67.77	2.21	13.47	2.11	13.41	1.03
	45	68.7	1.58	14.31	2.42	10.63	1.37
Control Samples	-	56.21	1.41	19.19	4.21	17.5	1.48

**Table 5:** Chemical compounds of EAF slag concretes.

Concrete	Chemical Compound (wt.%)							
	CaO	SO_3_	MgO	CaCO_3_	SiO_2_	Portlandite	Gypsum	C
Control	5	0	13	33	37.6	12.9	24.8	0
R1 15%	3	0	9	67	6	14	0	0
R1 30%	5.1	0	5.1	28.3	50.5	9.9	18.8	0
R1 45%	5	0	15	26	30	0	43	0
R2 15%	3	0	4	59.6	58.4	7.9	0	0
R2 30%	4	0	8.9	55.4	3	17.8	0	17.8
R2 45%	9.1	0	32.3	28.3	46	14	24	0
R3 15%	3	0	5	56	29	17	0	0
R3 30%	4	0	7	27	67	7	13	0
R3 45%	4	0	11.9	51.5	14.9	12.9	17.8	0

**Table 6:** Maximum CO_2_ uptake of concrete.

Concrete	Maximum CO_2_ Uptake (%)
Control	9.10
R1 15%	12.17
R1 30%	5.32
R1 45%	12.17
R2 15%	2.97
R2 30%	11.76
R2 45%	18.41
R3 15%	6.72
R3 30%	5.32
R3 45%	6.41

## Data Availability

The original contributions presented in this study are included in the article. Further inquiries can be directed to the corresponding author.
